# Predictors of dentists’ behaviours in delivering prevention in primary dental care in England: using the theory of planned behaviour

**DOI:** 10.1186/s12913-016-1293-x

**Published:** 2016-02-08

**Authors:** Huda Yusuf, Anna Kolliakou, Antiopi Ntouva, Marie Murphy, Tim Newton, Georgios Tsakos, Richard G. Watt

**Affiliations:** 1 Department of Epidemiology & Public Health, UCL, 1-19 Torrington Place, London, WC1E 7HB UK; 2 Institute of Psychiatry, Psychology & Neuroscience King’s College London, 16 De Crespigny Park, London, SE5 8AF UK; 3 Kings College Dental Institute, Denmark Hill, London, SE5 9RW UK

**Keywords:** TPB, Dentists, Prevention

## Abstract

**Background:**

To explore the factors predicting preventive behaviours among NHS dentists in Camden, Islington and Haringey in London, using constructs from the Theory of Planned Behaviour.

**Methods:**

A cross-sectional survey of NHS dentists working in North Central London was conducted. A self-completed questionnaire based on the theoretical framework of the Theory of Planned Behaviour was developed. It assessed dentists’ attitudes, current preventive activities, subjective norms and perceived behavioural control in delivering preventive care. In model 1, logistic regression was conducted to assess the relationship between a range of preventive behaviours (diet, smoking and alcohol) and the three TPB constructs attitude, subjective norms and perceived behavioural control. Model 2 was adjusted for intention.

**Results:**

Overall, 164 questionnaires were returned (response rate: 55.0 %). Dentists’ attitudes were important predictors of preventive behaviours among a sample of dentists in relation to asking and providing diet, alcohol and tobacco advice. A dentist was 3.73 times (95 % CI: 1.70, 8.18) more likely ask about a patient’s diet, if they had a positive attitude towards prevention, when adjusted for age, sex and intention. A similar pattern emerged for alcohol advice (OR 2.35, 95 % CI 1.12, 4.96). Dentists who had a positive attitude were also 2.59 times more likely to provide smoking cessation advice.

**Conclusions:**

The findings of this study have demonstrated that dentists’ attitudes are important predictors of preventive behaviours in relation to delivery of diet, smoking and alcohol advice.

## Background

One of the current challenges in healthcare is to reduce the burden of chronic diseases and tackle health inequalities to benefit populations and thereby decrease healthcare costs. Risk factors for chronic diseases include lifestyle behaviours such as smoking, poor diet and excessive alcohol use. Therefore, encouraging healthy behaviours among individuals may support the reduction in the incidence of chronic diseases [1].

Primary care provides opportunities for interaction between patient and health care professionals to deliver health promoting advice. Dental teams working in such settings are ideally positioned to provide evidence-based advice to their patients. It is recommended that dental teams ‘ask, assess, advice and assist’ in relation to risky health behaviours such as smoking, diet and alcohol consumption. However, evidence has shown that there is a gap between implementation of evidence-based advice and clinical practice. This is because of organisational, as well as clinician-related factors [2].

The proximal determinants of dentists’ preventive behaviours are most likely to be influenced by their attitudes, beliefs and values concerning the importance of and the ease of engaging in prevention. There is a wide range of psychological models and theories that provide an important framework for increasing our understanding of the determinants of health behaviours. One such model is the Theory of Planned Behaviour [3]. The Theory of Planned Behaviour (TPB) suggests that intention to perform an action is the most immediate predictor of behaviour. The strength of the intention, in turn is determined by three constructs: attitudes towards the behaviour, perceptions of personal control over the behaviour, and beliefs about social norms.

According to this theory, an individual is more likely to engage in a given behaviour, if he or she perceives that significant others endorse the behaviour and they wish to conform to this pressure, explained by the construct of social norms. Perceived behavioural control (PBC) describes the constraints as perceived by the individual. The general tenet holds that individuals are more disposed to engage in behaviours they feel are achievable and which are under their personal control.

Studies focusing on the behaviour of healthcare professionals demonstrated a high degree of variability in reported behaviour, related to the types of health professionals and the specific behaviours studied. In some studies, social norms were shown to be the most important predictor in predicting clinician’s behaviour, whereas others found the perceived behavioural control was the strongest predictor of intention among health professionals in providing advice about STDs in primary care [4, 5]. A systematic review of the relationship between intention and clinical behaviours (self-reported and observed) among a range of health professionals (doctors, nurses and pharmacists) identified 10 studies. Significant correlations between intention and self-reported behaviour were reported in four out of the five studies, but all correlations were low [6].

There have been few studies exploring behaviours among dentists by using the TPB. Bonetti et al., (2006) found that the two TPB constructs, namely attitude and perceived behavioural control, predicted behavioural intention to take intra-oral radiographs. Similarly, attitudes, subjective norms and perceived behavioural control predicted behavioural intention to place fissure sealants among a sample of dentists [7].

To date there is no research exploring predictors of multiple preventive behaviours (asking and advising on diet, tobacco and alcohol consumption) among dentists in primary dental care settings. To address this gap, the aim of this study was to assess whether dentists’ attitudes, subjective norms and perceived behavioural control predicted a range of preventive behaviours among NHS dentists in three areas in North Central London.

## Methods

A self-administered questionnaire survey targeting all NHS dentists in Camden, Islington and Haringey was conducted. The theoretical framework for the questionnaire was based on the Theory of Planned Behaviour [8, 9]. Findings from focus groups on prevention in primary dental care informed the development of themes in the questionnaire survey. In addition, validated questions from previous research in the dental literature were also included [10, 11]. The questionnaire was developed with the aim of collecting information on: the demography of dental performers, dental preventive activities provided (behaviours), their attitudes and beliefs about prevention, subjective norms, and perceived behavioural control.

Prior to the main study, the questionnaire was revised by the research team and was piloted and minor modifications were made. Ethical approval was obtained from Camden and Islington Community Research Ethics Committee (10/H072212).

The questionnaires and an accompanying letter, which provided an explanation of the study were sent to all NHS dentists, by mail. The participants were informed that their participation was voluntary and returning the self-administered questionnaire by mail was taken as consent. Private dentists, Hospital and Community Dentists were excluded from the study, as dentists working in General Dental Services within the National Health Services (NHS) provide the majority of dental care for the general population. The total potential sample size was 352. Those who did not respond were followed up by telephone calls and posted a second and then a third questionnaire a month later. Subsequently, non-responders in larger practices (more than 2 dental performers) were targeted and questionnaires were delivered and collected a week later, in person. In addition, dental performers who had not completed a questionnaire were encouraged to complete it in a training event, arranged by the research team.

The TPB constructs were measured using attitudes, subjective norms and perceived behavioural control.

Attitudes were measured with 3 questions:

“In the future, the General Dental Services should have a role to play in….?”

- smoking cessation
- alcohol advice
- dietary advice

(Response format: Strongly agree to strongly disagree, Likert scale)

Subjective norms were measured with two questions

“Most of my dental colleagues provide prevention for all their patients”. (Response format: Strongly agree to strongly disagree, Likert scale)

“My patients think as a dentist, I should use every opportunity to provide them with prevention.” (Response format: Strongly agree to strongly disagree, Likert scale)

Perceived behavioural control was measured with 2 questions

“I feel confident about practicing prevention if I wanted to” (Response format: Strongly agree to strongly disagree, Likert scale)

“For me to provide my patients with prevention is …” (Response format: very easy to very difficult, Likert Scale)

Intention was measured with one question.

“I aim to provide intention to all my patients” (Response format: Strongly agree to strongly disagree, Likert scale)

The six preventive behaviours were classified as *asking* about the behaviour and *providing advice* (direct intervention) in relation to diet, smoking and alcohol consumption.

Age, sex and country of qualification of dentists were used to describe the demography of the sample.

### Data analysis

The TPB constructs, were operationalized into scales by dichotomising responses around the median and then summing the items within each construct: attitudes, subjective norms, perceived behavioural control. Preventive behaviours including *asking about a patient’s behaviour* (diet, smoking and alcohol consumption) and *direct intervention* (provision of dietary, tobacco and alcohol advice) were also dichotomized around the median.

We predicted the different preventive behaviours, by using sequentially adjusted logistic regression models. In model 1, the relationship between behaviour (asking and intervening as outcomes) and (independent variables): attitude, subjective norms and perceived behavioural control was assessed, adjusted for age and sex. In model 2, the relationship between behaviour and the independent variables was adjusted for intention, age and sex. Data were analysed using StataSE 12.

## Results

A total of 164 dentists out of 300, participated in the study (response rate 55.0 %). The sample consisted of 60 % males, approximately a third of the sample were aged 30–39 years and 62.9 % of respondents had graduated from a UK university (Table 1).

**Table 1:** Demographic characteristics of the sample of dentists who completed the questionnaire

Characteristics	Number (*n* = 164)	Proportion (%)
Gender		
Male*	96	60.0
*Female*	64	40.0
Age Groups**		
* Under 30*	28	17.4
* 30–39*	53	32.9
* 40–49*	40	24.8
* 50–59*	28	17.4
* 60+*	12	7.5
Place of qualification***		
* UK*	88	62.9
* Europe*	30	21.4
* Other*	22	15.7

*n = 160, as there were 4 missing values

**n = 161, as there were 3 missing variables

***n = 140, as there were 3 missing variables

Examining preventive behaviour in relation to diet first revealed that a dentist was 3.73 times (95 % CI: 1.70, 8.18) more likely ask about a patient’s diet if they had a positive attitude towards prevention, when adjusted for age, sex and intention (Table 2). When the two models were adjusted for intention for both behaviours, the strength of the associations between asking (*p* = 0.001)/providing dietary advice (*p* = 0.009) and attitudes of dentists was attenuated though the results remained significant. Perceived behavioural control for both behaviours was initially a significant predictor for asking and providing dietary advice. However, when the models were adjusted, the effect of perceived behavioural control was mediated by intention for asking (*p* = 0.32) and providing dietary advice (*p* = 0.10). Subjective norms were not a significant predictor for either dietary behaviours.

**Table 2:** Logistic regression predicting preventive behaviours (asking about and providing diet advice) from the TPB variables

	Asking about dietary behaviour			Providing dietary advice		
	OR	CI	*P* value	OR	CI	*P* value
Attitude						
Model 1	4.69	(2.23, 9,86)	<0.001	4.10	(1.98, 8.50)	<0.001
Model 2	3.73	(1.70, 8.18)	0.001	2.82	(1.30, 6.11)	0.009
Subjective Norms						
Model 1	1.11	(0.57, 2.16)	0.75	1.83	(0.94, 3.56)	0.08
Model 2	0.73	(0.35, 1.51)	0.39	1.17	(0.56, 2.45)	0.68
Perceived Behavioural Control						
Model 1	2.37	(1.21, 4.64)	0.01	3.39	(1.71, 6.73)	<0.001
Model 2	1.46	(0.69, 3.11)	0.32	1.91	(0.89, 4.10)	0.10

Model 1: adjusted for age and sex

Model 2: adjusted for intention, age and sex

Exploring the relationships between asking about tobacco habits and providing tobacco advice, with attitudes, showed that overall dentists’ attitudes towards prevention had a significant effect on predicting these behaviours, when adjusted for age and sex (Table 3). However, when these models were adjusted for intention, the strength of the associations was attenuated for both asking about tobacco (OR: 2.21 95 % CI 0.89, 5.56, *p* = 0.09) and providing tobacco advice (OR: 2.59 95 % CI: 1.21, 5.53, *p* = 0.01). Perceived behavioural control was initially a significant predictor of tobacco-related preventive behaviours, however, the associations were fully explained when intention was accounted for. Similar to findings from dietary preventive behaviours, subjective norms were not a significant predictor for both asking and providing tobacco advice.

**Table 3:** Logistic regression predicting preventive behaviours (asking about and providing tobacco advice) from the TPB variables

	Asking about tobacco			Providing tobacco advice		
	OR	CI	*P* value	OR	CI	*P* value
Attitude						
Model 1	2.62	(1.09, 6.30)	0.03	3.65	(2.23, 9,86)	<0.001
Model 2	2.21	(0.89, 5.56)	0.09	2.59	(1.21, 5.53)	0.01
Subjective Norms						
Model 1	1.93	(0.88, 4.25)	0.10	1.52	(0.78, 2.97)	0.22
Model 2	1.46	(0.63, 3.41)	0.38	1.04	(0.50, 2.15)	0.92
Perceived Behavioural Control						
Model 1	2.66	(1.16, 6.09)	0.02	2.96	(1.49, 5.89)	0.002
Model 2	2.03	(0.80, 5.12)	0.13	1.91	(0.69, 3.11)	0.10

Model 1: adjusted for age and sex

Model 2: adjusted for intention, age and sex

Subsequently, the associations between asking about alcohol habits and providing advice were also explored separately with the four variables from the TPB: attitudes, subjective norms, perceived behavioural control and intention (Table 4). Dentist’s attitude remained a significant factor in predicting preventive behaviour, asking and intervening in relation to alcohol, after adjusting for intention. A similar pattern emerged for providing advice on alcohol consumption. However, when both models were adjusted for intention, a different pattern emerged between the two alcohol-related behaviours. There was a slight increase in odds ratios in terms of the effect of attitudes on asking about alcohol habits (OR: 2.35 95 % CI (1.12, 4.96), *p* = 0.03), whereas there was an attenuation in the provision of alcohol advice (OR: 2.95 95 % CI (1.15, 7.56), *p* = 0.02). Perceived behavioural control was significantly associated with providing alcohol advice, but when the model was adjusted for intention the results were insignificant. Likewise for the other preventive behaviours, subjective norms were not significantly associated with asking and providing advice on alcohol.

**Table 4:** Logistic regression predicting preventive behaviours (asking about and providing consumption alcohol advice) from the TPB variables

	Asking about alcohol consumption			Providing advice on alcohol intake		
	OR	CI	*P* value	OR	CI	*P* value
Attitude						
Model 1	2.20	(1.09, 4.44)	0.03	3.34	(1.36, 8.21)	0.009
Model 2	2.35	(1.12, 4.96)	0.03	2.95	(1.15, 7.56)	0.02
Subjective Norms						
Model 1	1.12	(0.58,2.14)	0.74	1.40	(0.60, 3.24)	0.43
Model 2	0.96	(0.48, 1.90)	0.90	1.13	(0.47, 2.72)	0.79
Perceived Behavioural Control						
Model 1	1.82	(0.94, 3.53)	0.07	2.78	(1.18, 6.58)	0.02
Model 2	1.72	(0.82, 3.62)	0.15	2.32	(0.89, 6.09)	0.09

Model 1: adjusted for age and sex

Model 2: adjusted for intention, age and sex

## Discussion

This study is the first to explore the prediction of a range of preventive behaviours among a sample of dentists in primary care using a psychological framework. Overall, one of the major predictors of preventive behaviours among this sample of dentists was their attitudes towards prevention, even when adjusted for intention. Subjective norms were unrelated to all six behaviours. Interestingly, the effects of perceived behavioural control were mediated by intention for the majority of behaviours.

We found consistent associations between the attitudes of dentists and their reported behaviours across a variety of preventive activities including diet, tobacco and alcohol. Therefore, the probability of a dentist engaging in preventive behaviour is enhanced in those with more positive attitudes towards preventive care. The effect of attitudes on a range of behaviours was attenuated when we adjusted for intention (asking and providing dietary, tobacco and alcohol advice). Perceived behavioural control was an important predictor for asking and intervening for all behaviours, but its role was fully explained when we adjusted for intention.

It is acknowledged that there are a limited number of studies which apply the TPB to understand behaviours of health professionals and the majority have focused on intention rather than reported behaviours [12]. Our findings are in partial agreement with other studies. A previous study assessing Scottish dentists’ behaviour in relation to placing fissure sealants found that all four constructs (attitude, subjective norms, perceived behavioural control, and intention) were important predictors of preventive behaviour [7]. On the other hand, some studies have shown that attitude was the most important predictor of intention in antibiotic prescriptions [13], in agreement with this study.

The relationship between attitudes and multiple preventive behaviours was attenuated when intention was introduced. Therefore, it is suggested that intention does not mediate the effect of attitudes completely and may be less important as a determinant of behaviour. This is also confirmed by the available literature. Studies have demonstrated that attitudes on their own can encourage certain behaviours, whereas other studies have shown that intention mediates the effects, which leads to behaviour change [14, 15]. This suggests that the roles of intentions and attitudes may vary depending on the situation and types of behaviours [16]. It could be that attitudes need to be translated into intention thereby influencing behaviour or it could be that attitudes are sufficient to influence behaviours directly [14]. A third alternative is that success in behaviour change creates positive attitudes – a process which is termed ‘enactive attainment’ [17]. In a cross sectional survey it is not possible for us to determine the direction of the relationship.

As noted in this study, perceived behavioural control was a predictor of the 6 preventive behaviours, without adjusting for intention. However, when intention was taken into account, the effect of perceived behavioural control was fully explained. Therefore, intention mediated the effect of perceived behavioural control to influence preventive behaviours. It is acknowledged that the association between perceived behavioural control and carrying out a behaviour is more complex and is influenced by the perceived amount of control we have over certain behaviours [18]. It is hypothesised that under circumstances of complete volitional control, perceived behavioural control would not have an impact on the intention-behaviour relationship [3]. However, if the behaviour is partially under volitional control, perceived behavioural control would moderate the relationship between intention and behaviour.

In relation to this study, subjective norms were not an important predictor of preventive behaviours. This compares favourably with the literature. Subjective norms have been shown to be the weakest component of predictor of intention [19] and it is suggested that the relationship between subjective norms is fully mediated by behavioural intentions [3]. However, this relationship is not consistent across studies. It could be due to inconsistencies in measuring subjective norms; many studies use a single item measure rather than employing multi-item scales [19].

Evidence has shown that there is a gap between the availability of evidence-based clinical guidelines and their implementation in daily practice [20]. Changing clinician behaviour is a complex process influenced by a number of factors including organisational, clinician and patient-related factors [21].

Furthermore, equipping clinicians with increasing knowledge and skills alone may not be sufficient in achieving behaviour change [22]. Michie et al, (2005) explored key theoretical constructs in implementation of evidence-based practice among clinicians and recommended that beliefs in capabilities and how to achieve changes may be additional factors that may need to be considered [23]. It is therefore necessary to develop a wider understanding of the fundamental factors that may influence dentists’ clinical practices and behaviours in order to improve healthcare delivery and health related outcomes for patients.

The findings from this study suggest that interventions aimed at changing dentists’ preventive behaviours should focus mainly on attitudes and to a lesser extent perceived behavioural control. This may entail providing dentists with training on the scientific evidence base on prevention and its potential benefits to patients, which may consequently result in a change of attitude towards the delivery of prevention.

While the majority of studies on dentists’ behaviours have focused on single behaviours, this study determined predictors of multiple preventive behaviours relevant to primary care dental practice.

While not being generalisable to the whole population of dentists, our results provide an insight into attitudes and behaviours of dentists working in primary dental care. The response rate was not high compared to the average of 60 %, expected for a postal questionnaire [24]. This means that our participants were not a representative sample. There is a risk of a bias response as only those who are interested in prevention having responded to the postal questionnaire. Furthermore, the questionnaires were based on self-report rather than observed behaviours and are therefore prone to bias.

## Conclusions

This study has shown that dentists’ attitudes are important predictors of a range of preventive behaviours including smoking, diet and alcohol advice. To encourage the implementation of evidence based guidelines on prevention in primary dental care, the findings from this study suggest that interventions should primarily focus on changing dentists’ attitudes. However, further research is required to identify further determinants which may be modifiable to influence dentists’ behaviors to improve patient care.
